# Diet’s impact on gut microbial assemblage in health and disease

**DOI:** 10.1172/JCI184319

**Published:** 2025-06-02

**Authors:** Carolina Koletic, Amanda Mrad, Anthony Martin, Suzanne Devkota

**Affiliations:** 1 F. Widjaja Inflammatory Bowel Diseases Institute and; 2 Human Microbiome Research Institute, Cedars-Sinai Medical Center, Los Angeles, California, USA.

## Abstract

The gut microbiome has been linked to everything from human behavior to athletic performance to disease pathogenesis. And yet, few universal truths have emerged regarding how the microbiome exerts its effects or responds to the host environment except for one: gut microbiota are exquisitely sensitive to human diets. What we eat from birth onward shapes our gut microbiome composition and function, and this is likely an evolutionarily conserved interaction that benefits the microbe and often the host. However, modern diets and lifestyles have created discordance between our slowly evolving human genome and rapidly adaptable microbiome, and have been implicated in the rise of chronic diseases over the past 75 years. Diet and microbiome interactions have been reviewed extensively, so here we focus on areas of microbiome research that have most illuminated natural and disruptive dietary forces over time in humans, and where we may have opportunities to restore the natural balance of host with microbes in our modern world.

## Introduction

The impact of diet on our gut microbiome composition and function cannot be underestimated. In fact, other than antibiotic perturbations, no other lever has been pulled more often than diet to manipulate the gut microbiome. These investigations have uncovered many reproducible findings that make up the pantheon of truths regarding how our microbiome is shaped by diet, including: (a) the most dynamic period of microbiota change is during the early period of life when the gut is exposed to different nutrition sources for the first time; (b) gut microbiota composition can change within 24 hours of a dietary change, but it is also resilient and often rebounds if the original diet is restored; and (c) that modern human microbiomes, especially those in urbanized regions with urbanized lifestyles, have diverged significantly from those of ancestral hominins and modern day populations in non-urbanized regions. Coupled with these, there are many associations with chronic diseases that have yet to be mechanistically proven in humans, but the combination of epidemiological observations, animal studies, and small human trials suggest we are standing at a precipice. Here, we explore diet and microbiome interactions across time scales and geography, highlighting what has changed in the modern food supply and dietary practices, possible mechanisms of action at the molecular level that may explain the microbiome’s contribution to disease, and also how we might make decisions for ourselves and our children that leverage the benefits of a gut microbiome for optimal health.

## Early microbiome assemblage in the GI tract

The composition of the initial infant microbiota after birth is primarily characterized by exposure to maternal stool via exposure to the perineum during vaginal birth, or maternal skin via cesarean section (C-section) (1, 2). The predominance of stool microbiota rather than vaginal microbiota in vaginally delivered babies was demonstrated prospectively in a proof-of-concept study, whereby seven infants born by C-section were selected to receive microbiota transplanted from either their own mother’s stool or vaginal swab. After three months, infants introduced to stool had more similar gut microbiota to that of control infants born vaginally than by C-section, more so than infants exposed to vaginal swabbing (3). This also suggests an evolutionary advantage of the proximity of the birth canal to the anus to ensure vertical transmission of microbiota to offspring. In a study of 41 pregnant women with vaginal and rectal swabs collected at six and eight months of gestation and two months postpartum, 16S rRNA sequencing revealed partial convergence of microbiomes at these sites. In this case, vaginal bacterial α diversity appeared to gradually increase, while rectal α diversity began to decrease, suggesting an alternative or additional way in which the proximity of the birth canal to the anus facilitates microbial transfer in humans (4). Indeed, acquisition of facultative anaerobes such as *E*. *coli* from the mother’s gut are suggested to be important for converting the semi-anaerobic infant gut to a fully anaerobic environment. These microbes preferentially consume the available oxygen, facilitating more rapid colonization of strict anaerobes, such as *Bifidobacteria* and *Clostridia,* that are needed to degrade human milk oligosaccharides (HMOs) and educate the developing immune system (5). Thus, maternal diet, insomuch as it shapes her own gut microbiome, can directly impact the sequence of colonization events in her newborn. While C-section–delivered babies are also initially colonized by oxygen-tolerant bacteria, these tend to be predominantly microaerophilic species of non-gut origin, resulting in different colonization dynamics at least through the first year of life compared with vaginally delivered babies (6).

**Figure 1:**
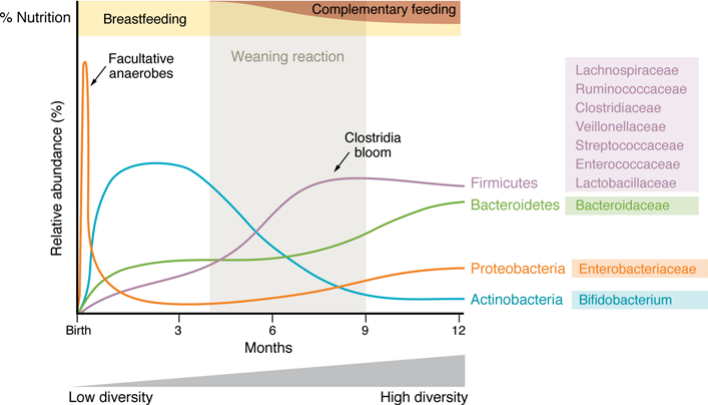
Microbial succession in the first two years of life and influences of different food exposures. The graph tracks phylum level changes in the first two years of life, highlighting the “weaning reaction.” The legend on the right focuses on the weaning reaction period, with finer taxonomic details on what genera increase during this period. Overall, the weaning reaction period is characterized by an increase in microbial diversity.

Vertical transfer of microbiota is important not only for gut colonization, but also for optimal immune development during a critical period in early life that coincides with weaning to solid foods. This so-called “weaning reaction” is defined by rapid diversification of the gut microbiome (Figure 1) and expansion of regulatory T cells (7). If colonization is restricted during this period, the immune development is stunted, leading to increased susceptibility to infection (8). Al Nabhani et al. also showed that germ-free (GF) mice have stunted immune systems but their immune development can be restored to levels of age-matched conventional mice if exposed to a healthy, conventional microbiota by 14 days of life; however, this restoration did not occur if exposed only after weaning after 28 days of life. If the weaning reaction is prevented, with antibiotics for example, then mice develop “pathological imprinting” characterized by susceptibility to inflammatory processes across the lifespan (7). The ensuing hypothesis is that the diet onto which an infant is weaned can prime their immune system for the rest of their lives via the gut microbiome, either beneficially or detrimentally. Sonnenburg et al. elegantly demonstrated in rodents that the fiber content of an infant’s diet may be critically important. Maternal consumption of low-fiber diets reduced gut microbiota diversity, and this reduced diversity was passed on to the offspring and perpetuated in subsequent generations if they were continued to be weaned onto a low-fiber diet. This pattern of reduced diversity was irreversibly altered within just two generations of low-fiber consumption (9). To further extend the aforementioned hypothesis, weaning onto low-fiber diets specifically may have maladaptive effects on the developing immune system due to poor diversification of the gut microbiome.

Prior to weaning, however, breast milk and/or formula make up the primary nutritional source for infants. Studying these nutritional sources on gut microbiome development has also been of intense interest. International guidelines recommend that infants be exclusively breastfed for the first six months of life, and then continue breastfeeding for up to 23 months of age or older, with introduction of appropriate complementary foods at 4 to 6 months of age (10–16). Multiple studies have shown that infants fed breast milk have distinct microbiota from those who are fed formula (17, 18), and most recently found to have distinct metabolomes as well (19). Furthermore, microbiome composition supported by breastfeeding is associated with decreased risk of allergy, asthma, and immune dysfunction compared with formula feeding (20, 21). Primary microbiota colonization determinants in breast milk include HMOs and secretory immunoglobulin A (SIgA). HMOs are non-lactose oligosaccharides produced by the maternal mammary glands that are not directly digested by the infant gut, but are utilized by specific bacteria, namely *Bifidobacterium* species and thus select for these genera (22). HMO selection of these bacteria is likely an evolutionarily conserved process, as numerous studies have demonstrated that early colonization by bacteria that have HMO-utilization genes dampen inflammatory responses from Th17 cells in favor of T regulatory responses (5, 23, 24). For example, *Bifidobacteria* have been shown to utilize HMOs to produce aromatic lactic acids that modulate CD4^^+^^ T cells and monocytes (25). Breast milk HMO diversity, however, varies widely across mothers and is influenced by their genetics (25) and diet (26), which makes it challenging to devise broad recommendations for optimizing HMO composition for infant health. Other important components of breast milk, such as SIgA, have only recently come to the forefront as important microbiome modulators in early life (26, 27). SIgAs are introduced through breast milk from birth and are also produced by intestinal epithelium around four weeks of age. SIgAs both agglutinate pathogens for immune clearance and also select for *Bifidobacteria*, *Streptococcus*, and *Lactobacillus* in the infant microbiome that help maintain the intestinal epithelial barrier and regulate immune development (28–33). Furthermore, deficiencies in SIgA can lead to expansion of antibody-unbound Enterobacteriaceae that has been shown to increase risk for necrotizing enterocolitis, a very serious intestinal disease that primarily affects premature infants (34, 35).

Further studies investigating breast milk components and their tripartite influence on gut microbiota colonization and immune development are needed to delineate mechanisms that may lead to tractable interventions to promote optimal gut health in infants. For example, infant formulas that include various HMOs have been shown to support microbial and metabolic signatures similar to those of exclusively breastfed infants, as well as reduced pathogen colonization and improved intestinal immune response (36, 37), which suggests a future for personalized infant formulas in mothers that may be deficient in a particular HMO.

In humans, diversification of the diet through the first introduction of solid foods recapitulates the gut microbiota diversification seen in rodent studies, with a shift away from the prominent *Bifidobacteria* and *Lactobacillus* and toward a more adult-like microbiome that includes *Ruminococcus*, *Prevotella*, *Clostridia*, *Bacteroides*, and *Dorea* species (38–40). When children are not exposed to diverse types of food as their gut develops, the microbiota remains immature, and this has been associated with stunted growth seen in severe acute malnutrition (41–44). However, human trials in children 12 to 18 years old with moderate acute malnutrition who were treated with supplemental microbiota-directed complementary food (MDCF) have shown promise in improving weight-for-length *z* (WLZ) scores, despite being lower in calories than traditional ready-to-use therapeutic foods (45). MDCFs contain a specific blend of fibers that result in expansion of *Faecalibacterium duncanei* (formerly *F*. *prausnitzii*), certain strains of *Segatella copri* (formerly *Prevotella* *copri*), and decrease in *Bifidobacterium longum*. These changes in microbiota were positively associated with proteins related to musculoskeletal growth and neurodevelopment and negatively associated with inflammation (46).

With the transition to a complete adult diet, the microbiome matures to resemble an adult microbiome by 3–5 years of age (18, 47). While the infant microbiome may not be directly predictive of adult microbiome composition, studies of early-life microbiome assemblage have most clearly demonstrated the profound importance of infant dietary exposures in coevolution between host and microbe as well as the critical role of our first microbes in educating the immune system and potentially imprinting increased or decreased risk of later-life disease.

## Diet shapes gut microbiomes across time and populations

Similar to investigations of the early-life microbiome, studies of ancestral microbiomes and microbiomes of geographically distinct populations allow us to examine how environmental factors such as diet shape the microbiome across larger time scales. For instance, the diets of ancient hominins from nearly 40,000 years ago were largely driven by seasonal and geographical constraints that rendered some populations as either predominantly carnivorous and others primarily plant based depending on local availability of food sources (48, 49). Today, some rural and geographically remote communities practice dietary habits similar to that of these ancestral humans (48–56). These present-day geographically native groups are largely hunter-gatherer and agrarian societies; this ancestral-like lifestyle is reflected in both the composition of their microbiomes and their incidence of chronic diseases, leading to the hypotheses that these factors are interrelated (51, 57–59). For example, a recurring observation in the gut microbiomes of individuals living in urban, industrialized areas includes reduced bacterial diversity relative to individuals living in rural areas that is coupled with increased incidence of metabolic, cardiovascular, and GI diseases (51, 54, 60, 61). Specifically, *S*. *copri*, a dominant species in rural populations and diminished in industrialized cohorts, has been associated with decreased risk for cardiometabolic disease (62, 63). Furthermore, as mentioned previously, certain strains of *S*. *copri* are associated with improved WLZ scores in malnourished children fed microbiome-supporting therapeutic foods (46).

To understand the trajectory of the ancestral microbiome to that of the modern human microbiome, it is important to contextualize how the diet of ancestral humans influenced their microbiomes. To make these comparisons, investigators have relied on human coprolites (fossilized feces) or dental calculi (calcified dental plaque) to map which microbial legacies have remained intact and which have been lost in the modern gut (64–69). Several studies that have broadly characterized genus-level microbial composition of paleofeces and dental calculi through 16S rRNA sequencing (64–66, 69); however, more recent work utilizing shotgun metagenomic sequencing has resolved deeper taxonomic identification of microbial communities, coupled with more in-depth interrogation of their genetic capacity (67, 68). Collectively, these data show that while clearly distinct from both rural and industrial modern human microbiomes, ancestral microbiomes most closely resemble modern rural microbiomes. A key genetic similarity between ancestral and modern non-industrialized microbiomes is the enrichment of transposases and mobile genetic elements that enhance adaptation to persistent variation in food bioavailability due to seasonal variation (67).

Recently, new taxonomic terminology, including BloSSUM (bloom or selected in societies of urbanization/modernization) and VANISH (volatile and/or associated negatively with industrialized societies of humans), have appeared in the literature due to the strong bidirectional associations of taxa in surveyed industrialized populations (51, 67, 70). Among the BloSSUM taxa, *Treponema succinifaciens —* a fiber-degrading spirochete — was found to be enriched in coprolite samples and many non-industrialized communities relative to industrialized communities (67, 70). Interestingly, an in-depth analysis of carbohydrate-active enzymes among ancient microbiomes, industrialized, and non-industrialized groups revealed that paleofeces and non-industrialized samples were enriched in fiber-degrading enzymes, specifically those targeting starch and glycans. This coincides with evidence of a traditional fiber-rich forager diet consisting of tubers and berries in both populations. In contrast, mucin-degrading enzymes were prevalent in industrialized samples largely from *Akkermansia muciniphila* (67). While generally regarded as a beneficial commensal, *A*. *muciniphila* can also promote intestinal barrier defects and food allergy in mice fed a fiber-free diet (71–73).

“Industrialization” and “urbanization,” terms often used interchangeably with “Western” diets, refer more to geographic regions globally or within a country, while “Western” refers to a certain dietary quantity and quality (caloric excess combined with high-fat/sugar/protein, and low-fiber) (74, 75). Typically, Western diets are associated with industrialized communities, while non-Western diets are associated with non-industrialized communities. As an epidemiological consequence, industrialized regions consuming Western diets have higher incidence of obesity and type 2 diabetes (76–78). Furthermore, a substantial body of literature has linked high-fat diets with decreased gut microbiota diversity (79), increased gut permeability associated with loss of microbiota-derived GLP-2 (80), and exacerbated gut inflammation (81, 82). The distinctions between industrialized and non-industrialized communities are increasingly becoming blurred with increased migration from rural, non-industrialized communities to nearby urban centers and associated adoption of local dietary habits. For example, employment-driven migrancy from rural communities in South Africa into urban centers promotes a transitional microbiome shifted in part due to adaptation to Western diets (70). This offers the opportunity to study how marked shifts in dietary pattern over an individual’s lifetime are reflected in their microbiome, and whether this has any impact on the risk for disease within an individual or increased frequency within a migrating population.

Indeed, studies have found that highly diverse gut microbiomes of non-industrialized, traditional communities who most closely resemble ancestral microbiomes have converged to resemble industrialized microbiomes upon migration to more industrialized societies, with a concomitant increase in disease incidence (60, 58, 83–86). The rate of inflammatory bowel diseases (IBD), for example, has increased over time in countries with previously low incidence, owing to more rapid adoption of Western dietary and lifestyle practices (86–89). IBD can manifest as either ulcerative colitis (UC) or Crohn disease (CD), with many subtypes therein. In historically non-Western and non-industrialized regions, UC rather than CD tends to be the prevalent form of IBD (90–94). With adoption of Western dietary and lifestyle practices, the presentation of IBD shifts to more CD cases than UC (87–94). Processed foods and high-fat, high-sugar diets have been implicated in the pathogenesis of CD (90). Furthermore, animal studies have demonstrated that deleterious changes in diet leading to loss of microbial diversity and ultimately extinction of taxa through successive familial generations is a growing reality (9).

Many populations throughout the world have diverged significantly from our ancestral microbiomes, which may have consequently impacted our overall health globally. Studies demonstrating extinction of ancestral bacterial species have led some to suggest that efforts to “re-wild” the gut microbiota through probiotic-like reintroduction of these lost species or fecal transplantation from non-industrialized populations could help reduce risk of chronic diseases. Aside from the ethical considerations of the latter and the unlikely success of either approach, dietary strategies may offer a more compelling opportunity. While we may not be able to restore our lost microbes, strategies such as eating foods in season from one’s native environment, avoiding highly processed foods, and increasing a diversity of fibers into the diet are relatively simple practices that the collective data provocatively suggest may promote a more non-urbanized microbiome and potentially reduce risk for chronic diseases. However, microbial “extinction” may be a form of adaptive evolution that is most optimized to modern diets and lifestyles, so we must consider the alternative hypothesis that “re-wilding” could create unanticipated health problems (95).

## Modern food additives inadvertently alter the gut microbiome

**Figure 2:**
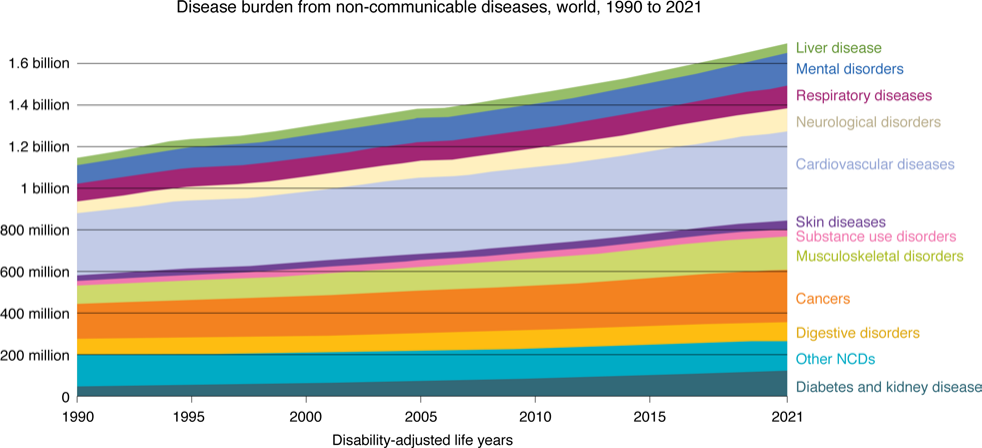
Rates of different chronic diseases over the past 30 years. Increases in chronic non-communicable disease (NCD) burdens ranged from 28% to 88% globally over the period of 1990-2021, coincide with increased adoption of Western dietary practices that are characterized by increased intake of fat and sugar, low intake of fiber, and inclusion of highly processed, additive-containing foods. Data sourced from the Global Burden of Disease Study 2021 (GBD 2021), Global Burden of Disease Collaborative Network, Institute for Health Metrics and Evaluation (IHME) (https://www.healthdata.org/research-analysis/library/global-burden-disease-2021-findings-gbd-2021-study). Used with permission. All rights reserved. Graph based on analysis of IHME data with minor processing by Our World in Data (https://ourworldindata.org/burden-of-disease).

The focus on understanding dietary effects on the gut microbiome has increased substantially over the past 20 years largely due to the observation that the rise in chronic diseases over the past 30+ years (Figure 2) coincides strikingly with increased adoption of Western dietary practices. Western diets are not only characterized as high-fat, high-sugar, and low-fiber, but are also highly processed and include an increasing number of additives to maintain shelf life and palatability. Recognition that gut microbes can serve as major mediators of host interactions with diet has generated multiple testable hypotheses of interactions of specific food additives with the host and gut microbiome.

Emulsifiers are widely used in processed foods to enhance texture and prolong shelf life by preventing the separation of water and oil components (96). Numerous studies indicate that emulsifiers can lead to intestinal inflammation in rodent models (96–100) and alter the gut microbiota; however, the changes in specific taxa vary across studies. Panyod et al. examined the effects of common dietary emulsifiers on intestinal permeability, metabolic biomarkers, and gut microbiota composition in mice. All four emulsifiers in the study, mono- and diglycerides (MDGs), lecithin, carboxymethylcellulose (CMC), and sucrose fatty acid esters altered microbiome composition, while MDG specifically caused an increase in circulating lipopolysaccharide (97). Similar findings were reported in a rodent study of P80 and carrageenan, also linking their consumption to thinning of the mucus layer and inflammation (98). Here, fecal microbiota transplantation from donor mice consuming P80 and CMC into GF mice was sufficient to induce inflammation in recipient mice, whereas GF mice did not exhibit inflammation when exposed to CMC and P80 in the absence of a microbiome (98). In a randomized, double-blind, placebo-controlled human study, exposure to CMC reduced microbial diversity, decreased short-chain fatty acid (SCFA) levels, and in some study participants, increased microbiota encroachment into the epithelium. Such changes suggest there would be an increased risk of inflammation, but in this study there was no significant change in fecal lipocalin, IL-17, or IL-8 (99). However, it is possible that the effects of these microbial changes in humans may act on non-immune pathways.

The most commonly used food dyes — Red 40, Red 5, Yellow 5, and Yellow 6 — are azo compounds that undergo bacterial transformation, with bacterial metabolic capacity and rates varying among species. This variability may explain why individuals exhibit varying sensitivities to dyes (101). Microbial metabolism of these chemicals is known to pose carcinogenic and inflammatory risks (102), and is suggested to occur extracellularly, due to the polar sulfur functional groups of the compound, in a pH-sensitive manner (103). Prior studies demonstrated that *Enterococcus* *faecalis*, *Enterococcus* *avium*, and *E*. *coli* utilize NADH-dependent electron transfers to break down the azo bonds in dyes (103, 104). Gut microbiota can also indirectly affect metabolism of food dyes via bacterial production of hydrogen sulfide (H__2__S). In this reaction, two H__2__S molecules donate four electrons to facilitate the cleavage of the azo bond (105). Bacterial H__2__S production can increase if directly consuming high levels of dietary sulfur sources in the form of cysteine or taurine, which tend to be higher in animal proteins, or indirectly by consuming high levels of saturated fats that increase endogenous production of taurine-conjugated bile acids (106). Promoting expansion of H__2__S-producing bacteria through the diet can thereby increase metabolism of food dyes. Red 40, a known carcinogen, and Yellow 6, have been found to induce colitis in conventional IL-23–overexpressing mice, but not in their GF counterparts. The functional unit in this case was 1-amino-2-naphthol-6-sulfonate sodium salt (ANSA-NA), a metabolite produced by both Red 40 and Yellow 6 when they are azo-reduced by bacteria. Red 3 and Blue 1, food dyes that do not have ANSA-NA, did not cause colitis in IL-23–overexpressing mice. Furthermore, direct treatment of rodents with ANSA-NA only induced colitis in conventional mice with intact microbiota but not in GF mice (107). Taken together, this may suggest that IBD patients with elevated IL-23 should actively reduce consumption of certain food dyes and animal products high in sulfur in order to suppress microbial metabolism of azo compounds.

Other widely used food colorants have also been studied. For example, titanium dioxide used to bleach foods has been found to disrupt SCFA production and induce inflammation (96, 108, 109). Additionally, Brilliant Black has shown efficient degradation by gut microbiota, facilitated by its azo bond containing four sulfur-electron-withdrawing groups, similar to Red 40 and Yellow 6 that contain two sulfur-electron-withdrawing groups (103). As a result, concerns have been raised about the impact of food dyes and by-products from microbial dye transformation on the gut epithelium and microbial communities (110).

Non-nutritive sweeteners (NNS) aim to combat obesity and diabetes by providing zero-calorie alternatives to sugar. Several studies have shown that NNS change the composition of the microbiome, and a subset of these studies have demonstrated that NNS may impair glucose tolerance via their induced changes in the gut microbiome (111–113). However, there are inconsistencies across studies. While Suez et al. found that sucralose and saccharin, but not aspartame, induced a potentially microbiome-dependent glucose intolerance in humans, Thomson et al. reported that sucralose did not impact the glucose tolerance in a double-blind randomized control trial in humans (111,114). Other studies suggest NNS may also increase antibiotic resistance. NNS can trigger bacterial SOS responses, a bacterial response to DNA damage characterized by the upregulation of DNA repair genes. This response can increase the rate of DNA mutation, gene transfer, and antibiotic resistance in bacteria, thus potentially increasing the risk of human disease (116, 117). However, it is important to note that quantity and frequency of NNS consumption matters, with “overconsumption” leading to potentially detrimental effects, while occasional use may be inconsequential to the microbiome. Additional work is required to clearly delineate dose-dependent effects across NNS in humans.

## Conclusions

Modern day medical and technological advances such as planned C-sections, ease of travel across continents, and food preservation to name a few, are conveniences in industrialized nations and urbanized centers that few could imagine living without. Indeed, technologies improving sanitization of water supplies, and vaccines, have increased the lifespan and decreased communicable diseases across entire populations. However, accumulating data over the past several decades have shown that rates of chronic diseases such as type 2 diabetes, autoimmune conditions, and neurodegenerative disease have increased significantly in urbanized regions compared with rural and agrarian regions, leading to the hypothesis that changes in dietary, social, and other environmental exposures may have benefited our convenience but harmed our health.

One concept explored is that our human genome is slow to evolve and has not yet adapted to modern lifestyles, and there are many examples in nature where this can result in significant reductions in populations and even extinction. Another compounding theory is that our gut microbiota adapts very quickly to changes in the environment. While evolutionarily we have become dependent on our gut microbiota to carry out essential metabolic and immune processes over the course of our life, our gut microbiota’s singular goal is to survive and multiply. So not only might we have discordance between our human genome and the environment, we may have created discordance between ourselves and our microbiome as well. In this Review, we explored a few key contexts where the data are compelling and where we may have opportunities to intervene, restore some of the beneficial relationships with our microbes that are health promoting, and try to make dietary and lifestyle decisions for children that support a robust microbiome in early life that will benefit subsequent generations.

## Version 1 06/02/2025

Electronic publication
